# Advanced MRI increases the diagnostic accuracy of recurrent glioblastoma: Single institution thresholds and validation of MR spectroscopy and diffusion weighted MR imaging

**DOI:** 10.1016/j.nicl.2016.02.016

**Published:** 2016-02-26

**Authors:** Tomas Kazda, Martin Bulik, Petr Pospisil, Radek Lakomy, Martin Smrcka, Pavel Slampa, Radim Jancalek

**Affiliations:** a International Clinical Research Center, St. Anne's University Hospital Brno, 656 91 Brno, Czech Republic; b Department of Radiation Oncology, Faculty of Medicine, Masaryk University, 625 00 Brno, Czech Republic; c Department of Radiation Oncology, Masaryk Memorial Cancer Institute, 656 53 Brno, Czech Republic; d Department of Diagnostic Imaging, Faculty of Medicine, Masaryk University, 625 00 Brno, Czech Republic; e Department of Diagnostic Imaging, St. Anne's University Hospital Brno, 656 91 Brno, Czech Republic; f Department of Comprehensive Cancer Care, Masaryk Memorial Cancer Institute, 656 53 Brno, Czech Republic; g Department of Comprehensive Cancer Care, Faculty of Medicine, Masaryk University, 625 00 Brno, Czech Republic; h Department of Neurosurgery, University Hospital Brno, Brno 625 00, Czech Republic; i Department of Neurosurgery, St. Anne's University Hospital Brno, Faculty of Medicine, Masaryk University, 625 00 Brno, Czech Republic; j Department of Neurosurgery, St. Anne's University Hospital Brno, 656 91 Brno, Czech Republic

**Keywords:** Glioma, Recurrence, Imaging sensitivity, Spectroscopy, Apparent diffusion coefficient

## Abstract

The accurate identification of glioblastoma progression remains an unmet clinical need. The aim of this prospective single-institutional study is to determine and validate thresholds for the main metabolite concentrations obtained by MR spectroscopy (MRS) and the values of the apparent diffusion coefficient (ADC) to enable distinguishing tumor recurrence from pseudoprogression. Thirty-nine patients after the standard treatment of a glioblastoma underwent advanced imaging by MRS and ADC at the time of suspected recurrence — median time to progression was 6.7 months. The highest significant sensitivity and specificity to call the glioblastoma recurrence was observed for the total choline (tCho) to total N-acetylaspartate (tNAA) concentration ratio with the threshold ≥ 1.3 (sensitivity 100.0% and specificity 94.7%). The ADCmean value higher than 1313 × 10^− 6^ mm^2^/s was associated with the pseudoprogression (sensitivity 98.3%, specificity 100.0%). The combination of MRS focused on the tCho/tNAA concentration ratio and the ADCmean value represents imaging methods applicable to early non-invasive differentiation between a glioblastoma recurrence and a pseudoprogression. However, the institutional definition and validation of thresholds for differential diagnostics is needed for the elimination of setup errors before implementation of these multimodal imaging techniques into clinical practice, as well as into clinical trials.

## Highlights

• For an effective salvage treatment, an accurate diagnosis of GBM recurrence is essential.
• The standard structural MRI has limited sensitivity and specificity to distinguish GBM progression.
• GBM recurrence is characterized by the ADCmean value ≤ 1313 × 10^− 6^ mm^2^/s and the tCho/tNAA ratio ≥ 1.3.
• An institutional definition of thresholds is needed, if advanced imaging should be used accurately in clinical practice.

## 1 Introduction

The critical biological characteristic of a glioblastoma (GBM), the most frequent and serious primary brain tumor in adults is an inevitable progression after standard therapy with the median of 6.9 months (Dusek et al., 2014, Stupp et al., 2005). Tumor recurrence develops in almost all patients despite the aggressive standard first line treatment, which comprised of radiotherapy and temozolomide usage (RT and TMZ) (Stupp et al., 2005). GBM recurrence, however, has often similar radiologic characteristics on conventional MRI as therapy-related changes, like a pseudoprogression (PsP). Thus, the early and accurate diagnosis of GBM relapse constitutes to be an important clinical need, especially when more and more potentially active drugs are currently being investigated for salvage treatment.

In comparison with standard structural MRI techniques, advanced imaging methods, such as diffusion-weighted imaging (DWI) with apparent diffusion coefficient (ADC) mapping, and the proton MR spectroscopy (MRS), allow much deeper and still non-invasive insight into the interpretation of brain lesions, resulting in greater specificity of diagnostic imaging (Ahmed et al., 2014, Bulik et al., 2015, Kao et al., 2013, Roy et al., 2013). In our previous report of the consecutive series of 24 patients with GBM, we described significant differences in ADC and MRS data between those with GBM relapse and PsP after standard RT and TMZ treatment (Bulik et al., 2015). However, thresholds with higher statistical power and intra-institutional validation have been required before these methods can be implemented into our institutional imaging protocols on a routine basis and used in the decision-making process. In this report, we present our final results of this prospective study with extended number of patients, as well as the results from an independent retrospective intra-institutional validation.

## 2 Methods

### 2.1 Characteristics of patients

Patients suitable for this study included the ones with histologically proven GBM after gross total resection, as stated by an early post-surgery MRI examination, who underwent the standard adjuvant treatment consisting of concurrent RT (dose 60 Gy) and TMZ followed by adjuvant TMZ alone (Stupp et al., 2009). The standard follow-up imaging protocol at our institution is the classic structural MRI after 6 weeks and every 3 months thereafter. Patients were eligible for study enrollment when suspected radiographic progression on the structural MRI was found based on the neuro-radiologist's discretion. After they signed an informed consent, the patients underwent the investigational advanced MRI, namely MRS and DWI. The final evidence of the disease status was realized by means of biopsy/resection or early repeated structural MRI depending on the clinical situation, patient's performance status, de facto his or her best interest. The advanced imaging protocol was approved by the Institutional Review Board of the St. Anne's University Hospital Brno. Patients previously described in our initial analysis are also included in the current study cohort (Bulik et al., 2015). The validation cohort consisted of the independent series of previous patients with GBM treated by surgery and adjuvant RT and TMZ, who underwent MRS and DWI/ADC exams according to the same protocol. Initially, the derived thresholds for pertinent MRS spectra and ADCmean values were subsequently applied to predict a GBM recurrence or treatment related changes, such as PsP and radionecrosis.

### 2.2 MR data acquisition

The advanced MRI examination was performed at 3.0T clinical MR scanner (GE Medical Systems Discovery MR750), following the same setup parameters as in our initial report (Bulik et al., 2015). MRS was focused on gadolinium-enhanced lesions suspected of recurrence by means of the chemical shift imaging (CSI) technique in two orthogonal planes respecting the long axis of the lesion and the proximity to structures increasing noise in MR spectra (point-resolved spectroscopy sequence — PRESS, TR/TE 1800/144 ms, 16-cm FOV, 15-mm slice thickness, and voxel size 10 × 10 × 15 mm^3^). Automatic prescanning was performed prior to each spectroscopic scan to ensure adequate water suppression. The MR spectra of all measured voxels were automatically post-processed for each patient by LCModel version 6.3 (Provencher, 2001). The output of MRS processing by LCModel were the concentrations of N-acetylaspartate and N-acetylaspartylglutamate (tNAA), choline-containing compounds (tCho), total creatine (tCr), lipids at 0.9–1.3 ppm, and lactate (Lac). Afterwards, the ratios of the metabolite concentrations (tCho/tNAA, tCho/tCr, tNAA/tCr, Lac + Lip1.3/tCr, Lac + Lip0.9–1.3/tCr) were calculated and visualized by jSIPRO 1.0 beta version (Jiru et al., 2013). The signal-to-noise ratio for each MR spectrum and an error map showing the error in a measured concentration for each metabolite were calculated by using the jSIPRO software. The concept of error images in jSIPRO was developed to help rejection of low quality spectra (Jiru et al., 2006). From the voxels covering gadolinium-enhancing region, these with the signal-to-noise ratio > 3 and the error of measured metabolite concentrations < 20% where selected and arranged based on the value of the Cho/NAA ratio. The voxels with the highest Cho/NAA ratio were selected for further analyses.

An axial echo planar SE sequence (TR/TE 6000/100 ms), 5-mm slice thickness, and diffusion gradient encoding in three orthogonal directions (*b* = 0 and 1000 mm^2^/s, and 240-mm FOV) were utilized for DWI imaging. ADC maps were calculated using the OsiriX software version 6.0.2 64-bit (Pixmeo SARL, Switzerland) with the ADC Map Calculation plugin version 1.9 (Stanford University). The mean ADC value (ADCmean) of the voxel corresponding to the measured MRS voxel was calculated.

### 2.3 Data analysis

The optimal diagnostic cut-offs and the description of their sensitivity and specificity for the final diagnosis of recurrence were derived from the receiver operating characteristic (ROC) analysis with the area under the ROC curve (AUC) for distinguishing between the two diagnostic groups (GBM relapse and PsP). Fisher's exact test for categorical data and Mann–Whitney *U* test for continuous variables were used to estimate the significance of measured differences. Censoring the patients who were lost for the follow up, the overall survival was defined as the time elapsed between the GBM diagnosis and death from any cause. The time to progression was measured since the end of RT and TMZ with suspected progression at structural MRI as the event of interest. The probability value *p* < 0.05 was considered statistically significant in all tests. All statistical evaluations were performed using the statistical software Statistica 12 (StatSoft, Inc.).

## 3 Results

### 3.1 Study patient characteristics

Between May 2013 and March 2015, the total of 39 patients (median age 51, 72% men) with suspected GBM progression on the structural MRI was prospectively included into this study. The basic characteristics of patients are summarized in Table 1. The median time to suspected progression and the median overall survival were 6.7 months (95% CI 2.9–9.6) and 14.5 months (95% CI 12.9–17.4), respectively. The final diagnosis was established by a biopsy in 26 patients (67%) and by follow-up imaging in 13 patients (33%). The diagnosis of a GBM recurrence yielded in 29 patients (75%) with the rest having PsP. No case of radionecrosis was found in our cohort of patients.

### 3.2 Advanced imaging characteristics

**Fig. 1:**
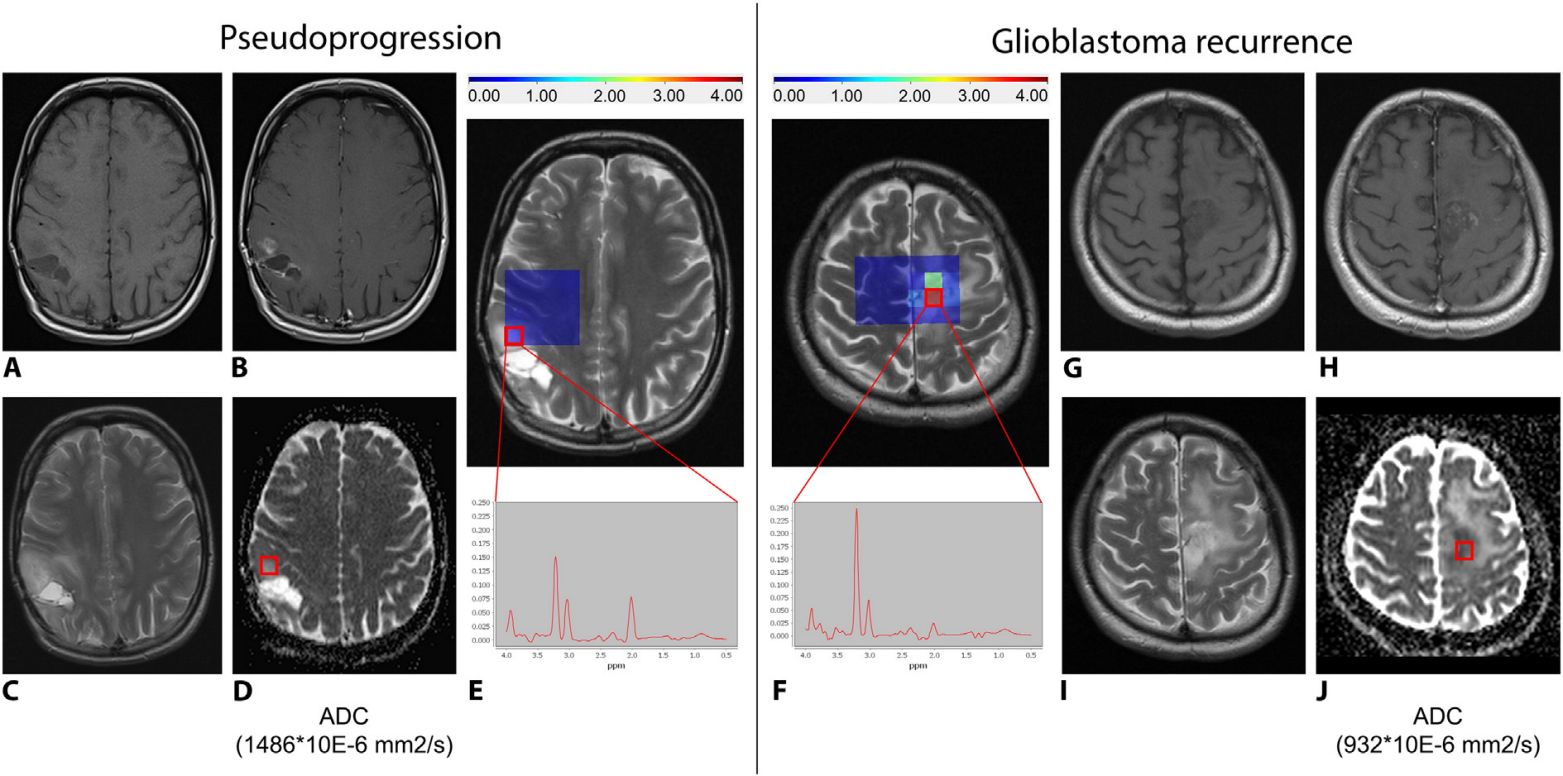
The pseudoprogression and glioblastoma recurrence findings on MRI. Representative MRI examples of the pseudoprogression (A–E) and glioblastoma relapse (F–J): (A) + (G) show T1WI, (B) + (H) show T1WI with gadolinium, (C) + (I) show T2WI, (D) + (J) show the ADC maps with marked VOI and corresponding ADCmean values, and (E) + (F) show the proton MR spectroscopy maps focused on tCho/tNAA concentration ratio with marked VOI, corresponding values and spectra. The MR spectra illustrate concentrations of the main metabolites within the selected voxel. The color scale for the tCho/tNAA ratio corresponds to the color map shown for the MRSI grids overlaid on top of the MR images. The signal-to-noise ratio of the presented MR spectra is 4.98 in pseudoprogression and 4.86 in glioblastoma recurrence as well as the error of tCho/tNAA concentration 7% and 4%, respectively.

The values of metabolite concentration ratios are summarized in Table 2, the percentage distribution of patients in Table 3, and typical imaging findings are presented in Fig. 1. The mean and standard deviation of the signal-to-noise ratios of MR spectra in the analyzed voxels was 4.75 ± 0.80. The average number of voxels with acceptable spectra quality per patient was 3.75 ± 1.13 and varied based on the proximity of the skull and a resection cavity as the most significant noise-conducting factors.

A significant difference in the tCho/tNAA and tNAA/tCr ratios was found between the GBM relapse and PsP. The GBM relapse was characterized by the tCho/tNAA ratio ≥ 1.3 with sensitivity of 100% and specificity of 94.7% (*p* < 0,001). All patients with GBM recurrence had the value of tCho/tNAA above this cut-off; yet, there were still 5.3% (1/19) lesion assigned as PsP reaching the same tCho/tNAA cut-off as GBM recurrence. Another metabolite ratio with statistical significance was tNAA/tCr characterized by the threshold ≤ 0.7 for calling the GBM recurrence with sensitivity of 96.6% and specificity of 94.7% (*p* < 0,001). There were 93.2% (55/59) lesion considered as the GBM recurrence that had the tNAA/tCr values below the cut-off, just as 5.3% (1/19) as PsP.

The calculated ADCmean values were significantly lower in the GBM relapse group than in the PsP group (*p* < 0.001), with the cut-off of 1313 × 10^− 6^ mm^2^/s (sensitivity 98.3% and specificity 100.0%). Ninety-eight percent of patients with the GBM relapse had the ADCmean ≤ 1313 × 10^− 6^ mm^2^/s while all the patients with PsP had the ADCmean > 1313 × 10^− 6^ mm^2^/s.

### 3.3 Characteristics of the patients in the validation cohort

The basic characteristics of the patients in the validation cohort are summarized in Table 1 and are balanced with the study cohort. Their pertinent metabolite concentrations and ADCmean values are reported in Table 4 together with the level of success in the prediction of diagnosis by each measured characteristic. The tCho/tNAA ratio assigned diagnosis correctly in 15/16 (94%) patients, the ADCmean value in 15/16 (94%) patients, the concentration ratio of tNAA/tCr in 13/16 (81%) patients, while tCho/tCr, Lac + Lip 1.3/tCr and Lac + Lip 0.9–1.3/tCr only in 8/16 (50%) patients. These results confirm expected specificity for the measured MR characteristics. The combination of tCho/tNAA and ADCmean led to the highest accuracy while establishing the final diagnosis.

## 4 Discussion

The accurate and timely identification of a tumor relapse is the most essential prerequisite of an efficient salvage therapy emphasizing the importance of precise assessment of the response to the initial treatment. Well-known difficulties with distinguishing between a GBM recurrence and treatment related changes caused by the administration of concomitant RT and TMZ (pseudoprogression) or angiogenesis inhibitors (pseudoresponse) (Hygino da Cruz et al., 2011, Chakravarti et al., 2006) are already expressed in the current RANO (Response Assessment in Neuro-Oncology) criteria (Wen et al., 2010). Nevertheless, the evolution of the response criteria that is a continuing process and implementation of advanced MRI methods is expected, most likely by MRS and DWI because of their high availability. Especially for these advanced MRI methods, the standardization of MRI protocols is needed in order to be used optimally in the evaluation of results from multicentric studies. The topicality of this need is expressed by the current consensual recommendations for the standard brain tumor imaging protocol in clinical trials published in September 2015, which already include pre-contrast, axial 2D, 3-directional DWI (Ellingson et al., 2015). Before similar recommendations for other advanced MRI modalities are established, centers utilizing these methods to resolve ambiguous findings in the classic structural MRI should develop their own thresholds and cut-offs for a valid image description.

The MRS seems to be a promising method that is complementary to the widely used structural MRI and can be used to increase the diagnostic accuracy of the brain tumor imaging protocol. The results of this study proved very high sensitivity and specificity of the tCho/tNAA concentration ratio (100.0% and 94.7%, respectively) for a non-invasive differentiation between a GBM recurrence and PsP. The underlying pathophysiology of the MRS observations is well described especially from the experience with glioma grading (Bulik et al., 2013). Choline represents the marker of cell membrane integrity and turnover and is associated with the presence of an increased tumor cell proliferation, while NAA is the marker of the density and viability of neurons. Thus, their mutual ratio forms the best approach when using the MRS for brain tumor diagnostics. The result of the MRS, though, depends on the type of the MR scanner, specific acquisition setup parameters, and is very sensitive to the proximity of FOV to the surrounding structures decreasing signal/noise ratio of the brain spectra (i.e. bone). As the spectrum quality is also highly influenced by the personal experience of a radiologist, it is useful to establish an institutional protocol with the adjustment of thresholds and cut-offs for the main metabolite concentrations measured by the MRS. Moreover, there is the significant heterogeneity of glial tumor tissue and many suspected recurrences are localized in close proximity to the bone increasing noise in the acquired MR spectra. Thus, it is useful to perform the MRS by means of a Spectroscopic Imaging technique in two orthogonal planes covering most of the enhancing brain regions. We faced all the described difficulties in MRS voxel analysis since the patients of our cohort underwent gross total resection (GTR). This patients' selection was needed, whether we wanted to analyze the ability of MRS and ADC maps to distinguish strictly between the pseudoprogression and tumor recurrence with no bias by a residual tumor. The probability to achieve GTR is higher in the case of small and superficial tumors located in the proximity of the skull. In addition, the majority of gadolinium-enhancing lesions were heterogeneous, irregular in shape, and small due to a frequent follow-up that further underline necessity to use the Spectroscopic Imaging technique and strict voxel selection. Compare to our methodology, Single Voxel MR spectroscopy is often used in published studies but it is highly influenced by the partial volume effect where the obtained spectra are distorted by surrounding tissue (Lee et al., 2013).

There are several studies focusing on MRS for the purpose of differentiating glioma recurrence from treatment related changes. The current meta-analysis by Zhang et al., involving 18 studies and 455 patients, showed only moderate sensitivity and specificity of the tCho/tNAA ratio (88% and 86%, respectively) for differentiating a recurrent glioma from radiation necrosis (Zhang et al., 2014). However, the late treatment related changes of RT and TMZ have some similar histopathologic features as a high-grade glioma relapse (e.g. the presence of necrosis) that can lead to a decreased accuracy of MRS diagnostic and can explain our superior results because we observed only patients with PsP. Moreover, most studies reviewed by Zhang and colleagues used Single Voxel MRS where average values from larger voxels are produced. The analysis of individual small voxels may also explain higher sensitivity and specificity observed in the presented study. Nevertheless, as we can expect lower sensitivity and specificity of the MRS in the general diagnostic protocol for differential diagnostic in neuro-oncology, we agree with the recommendation of Zhang et al. to combine MRS with other multimodal imaging methods. For example, that would be definitely the case of the patient number 12 from our validation cohort where conflicting results of MRS and DWI were presented. Fortunately, this patient was able to undergo biopsy validation confirming the tumor recurrence. Otherwise, close follow-up with early repeated imaging studies would be indicated.

Diffusion-weighted imaging describes changes in water diffusivity mainly as the function of changes in cell density. The diffusion changes can be quantified by the ADC. Generally speaking, decreased diffusivity is the consequence of an enhanced tumor cell proliferation and is reflected by the water diffusion restriction that lowers ADC values. In the present study, the evaluation of mean ADC values led to the highest sensitivity and specificity with the cut-off of 1313 × 10^− 6^ mm^2^/s in distinguishing between a GBM recurrence and PsP that was proved in our validation cohort of patients. For all patients with PsP from this study cohort, the ADC mean value was > 1313 × 10^− 6^ mm^2^/s. However, evaluating the diagnostic quality of DWI in differentiating glioma recurrence from radiation necrosis, the current meta-analysis by Zhang et al. from April 2015 pooled and weighted data from 284 patients, and showed only moderate diagnostic performance in differentiating the glioma recurrence with sensitivity of 82% (95% CI: 75,87) and specificity of 84% (95% CI: 76,91) (Zhang et al., 2015). In the present study, the higher sensitivity and specificity observed can be explained by the same way as in the case of the MRS mentioned above — treatment related changes represented exclusively by PsP with no case of radionecrosis.

The lack of radionecrosis in our cohort can be explained by a time factor. The aim of our study was to describe early MRI changes after oncology treatment; however, radionecrosis is more often related to the late effect of radiotherapy. Regardless of low radionecrosis incidence in selected group of patients after RT and TMZ (9.3% of patients), Ruben with co-authors described that mean interval from the completion of radiotherapy to the diagnosis of radionecrosis was 11.6 months in the cohort of 426 patients treated for glioma (Ruben et al., 2006). The lack of radionecrosis may be also related to the gross total extent of resections and generally less aggressive strategy in delivery of radiotherapy (normalization to 95% of prescribed dose, strict limitations of Dmax, less generous target definition strategy with reference to RTOG rather than to EORTC approach).

This study has also some inherent limitations. The fact that a biopsy for proving the final diagnosis (recurrence vs. treatment related changes) was missing in 33% of patients, as their best interest was reflected, may prevent the deeper explanation of the observed metabolite concentrations or the ADC data. It may be assumed that some patients develop the overlapping imaging features of both PsP and early GBM recurrence at the same time, which lowers the tCho/tNAA ratio and increases the ADCmean value due to the predominance of initial PsP changes. On the other hand, patients with PsP are more often those with a favorable prognosis and the concurrent presence of PsP and early GBM progression is not a case of all patients with PsP. Moreover, the use of biopsy samples may also be difficult to interpret because of the above mentioned tissue mixture, as well as the post radiotherapy changes. It means that the single target stereotactic needle biopsy of the lesion suspected of a tumor recurrence may be inaccurate (Hygino da Cruz et al., 2011). Patients who are not able to undergo tumor resection or at least biopsy validation may most benefit from the non-invasive nature of advanced MRI methods and, in clinical practice, they may be candidates for further imaging studies including MR perfusion or positron emission tomography examination. With this consideration, we can agree with the recommendation of Zhang et al. to combine DWI imaging results with other multimodal imaging methods (Zhang et al., 2014, Zhang et al., 2015). Thus, combination of ADC values and metabolite concentrations measured by MRS could produce a single prediction with a significant clinical impact; however, other studies with more patients included are needed for valid recommendations.

## 5 Conclusion

In conclusion, there is the increasing evidence for routine utilization of advanced MRI methods such as DWI and MRS in brain tumor imaging protocols. This study has proved that the combination of ADCmean values ≤ 1313 × 10^− 6^ mm2/s and the tCho/tNAA concentration ratio ≥ 1.3 have the high validity for a non-invasive differentiation between a GBM recurrence and pseudoprogression. However, institutional definition and validation of the thresholds of the advanced MR methods is needed in order to implement the multimodal imaging into routine clinical practice, as well as clinical trials.

## Conflicts of interest

The authors declare that they have no conflicts of interest.

## Funding/Acknowledgments

The study was supported by the Czech Ministry of Health Grants No. NT14120-3/2013 and NT14600-3/2013, as well as the European Regional Development Fund, Project FNUSA-ICRC (No. CZ.1.05/1.1.00/02.0123), and MH CZ-DRO (MMCI, 00209805).

## Figures and Tables

**Table 1:** Basic characteristics of the study cohort: T = temporal, F = frontal, P = parietal, O = occipital, F–P = frontoparietal, 3D-CRT = Three-Dimensional Conformal Radiotherapy, IMRT = Intensity-Modulated Radiotherapy.

Basic characteristics	*Study cohort* *n* = 39	*Validation* *n* = *16*	*p*
*Age at initial diagnosis* (*years*)			
Median	51	54	0.7
Range	29–66	35–64	
*Sex* (*n*)			
Men	28 (72%)	10 (63%)	0.5
*GBM location* (%)			
T/F/P/O/F-P	38/29/19/5/9	34/31/14/7/14	0.7
*Radiotherapy*			
Median dose (Gy)	60	60	0.9
Technique 3D-CRT/IMRT (%)	30/70	40/60	0.8
*Cycles of adjuvant TMZ*			
Median	6	6	0.9
Range	1–12	4–10	
*Time to graphic progression* (*months*)			
Median (95% CI)	6.7 (2.9–9.6)	6.1 (4.8–8.8)	0.8
*Diagnosis validation*			
Biopsy/subsequent imaging (%)	67/33	75/25	0.6
*Final diagnosis*			
Tumor recurrence	29 (75%)	12 (75%)	1
Pseudoprogression	10 (25%)	4 (25%)	
*Overall survival* (*months*)			
Median (95% CI)	14.5 (12.9–17.4)	14.0 (13.1–15.2)	0.8

**Table 2:** Calculated cut-offs for the diagnosis of a tumor recurrence with related sensitivity and specificity for the most important concentration ratios of the metabolites measured by MRS and for ADCmean.

Metabolite	AUC (95% CI)	*p*	Cut-off for recurrence	Sensitivity	Specificity
tCho/tNAA	0.993 (0.978; 1.000)	< 0.001	≥ 1.3	100.0	94.7
tCho/tCr	0.691 (0.539; 0.843)	0.013	≥ 0.7	74.6	63.2
tNAA/tCr	0.949 (0.873; 1.000)	< 0.001	≤ 0.7	96.6	94.7
Lac + Lip 1.3/tCr	0.714 (0.559; 0.868)	0.003	≥ 1.6	76.3	68.4
Lac + Lip 0.9–1.3/tCr	0.723 (0.568; 0.879)	0.004	≥ 2.0	78.0	68.4
ADCmean [10^− 6^ mm^2^/s]	0.998 (0.993; 1.000)	< 0.001	≤ 1313	98.3	100.0

**Table 3:** The percentage distribution of patients with the pseudoprogression and glioblastoma recurrence as the function of calculated cut-offs.

		Pseudoprogression (n = 19)	Recurrence (n = 59)	*p*
*tCho/tNAA*	< 1.3	18 (94.7%)	0	< 0.001
≥ 1.3	1 (5.3%)	59 (100.0%)
Median (min; max)		0.74 (0.33–1.77)	2.13 (1.35–9.60)	< 0.001
*tCho/tCr*	< 0.7	11 (57.9%)	15 (25.4%)	0.013
≥ 0.7	8 (42.1%)	44 (74.6%)
Median (min; max)		0.64 (0.28–1.37)	0.89 (0.44–2.83)	0.013
*tNAA/tCr*	> 0.7	18 (94.7%)	4 (6.8%)	< 0.001
≤ 0.7	1 (5.3%)	55 (93.2%)
Median (min; max)		0.99 (0.28–1.59)	0.41 (0.11–0.96)	< 0.001
*Lac* *+* *Lip 1.3/tCr*	< 1.6	12 (63.2%)	14 (23.7%)	0.004
≥ 1.6	7 (36.8%)	45 (76.3%)
Median (min; max)		1.13 (0.07–10.65)	2.69 (0.40–15.63)	0.005
*Lac* *+* *Lip 0.9–1.3/tCr*	< 2.0	13 (68.4%)	13 (22.0%)	< 0.001
≥ 2.0	6 (31.6%)	46 (78.0%)
Median (min; max)		1.33 (0.08–12.35)	3.26 (0.54–17.42)	0.004
*ADCmean*	> 1313	19 (100.0%)	1 (1.7%)	< 0.001
≤ 1313	0	58 (98.3%)
Median (min; max)		1372 (1317–1476)	1155(756–1344)	< 0.001

**Table 4:**
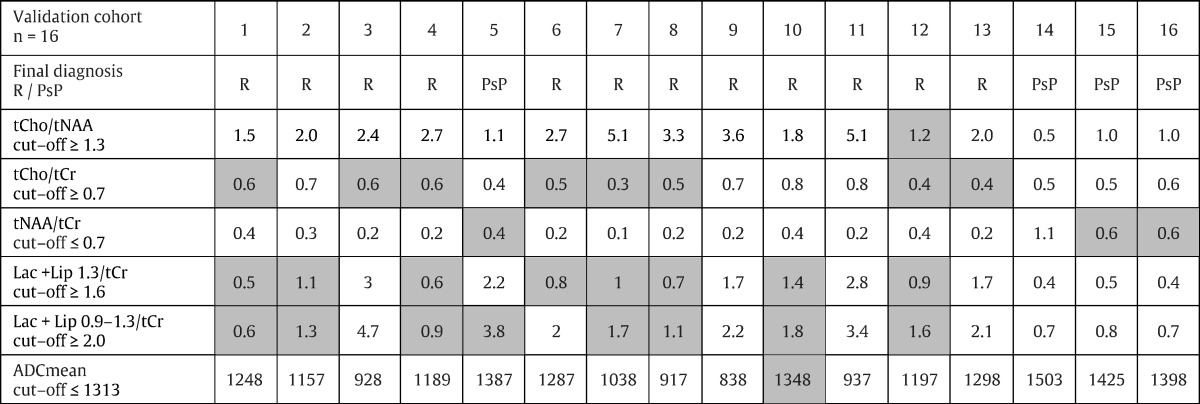
The validation of the calculated metabolite concentration ratios and ADCmean cut-offs for the GBM recurrence by the respective cohort of patients. The gray color indicates discrepancy between the predicted value and the real measured value. R — recurrence, PsP — pseudoprogression.
